# Faf2 is required for neural differentiation in embryonic neural progenitor cells

**DOI:** 10.3389/fcell.2026.1935883

**Published:** 2026-08-26

**Authors:** Anneke Dixie Kakebeen, Leon Dunphy, Heather K. Hazen, Lee A. Niswander

**Affiliations:** Department of Molecular, Cellular, and Developmental Biology, University of Colorado Boulder, Boulder, CO, United States

**Keywords:** cell fate decisions, ER stress, FAF2, neural differentiation, neural progenitor cells

## Abstract

Neural progenitor cell differentiation is a complex process requiring the proper integration of instructive and permissive factors. Instructive cues including signaling molecules and transcription factor networks have been well studied in this context, but permissive factors such as cell homeostasis have not. Cell homeostasis is critical to support the health and stability of a cell and enable the cell to act on instructive differentiation cues. Our study investigates a homeostasis protein, FAF2, and its function in neural progenitor cells. FAF2 is an adaptor protein involved in endoplasmic reticulum (ER) associated degradation to remove misfolded proteins and restore ER homeostasis. Here we show that knocking out *Faf2* in neural progenitor cells results in increased ER stress signature at the protein and transcription level, indicating a conserved functional role in neural progenitor cells. Induced neural differentiation of FAF2 deletion cells shows a failure of neurite development but RNA-seq indicates genes that support neural differentiation are induced. Reducing ER stress in FAF2 knockout cells with a small molecule inhibitor can rescue neural differentiation, providing evidence that excess ER stress contributes to the inhibited differentiation. Taken together, these results reveal that FAF2 is a critical protein in neural progenitor cells for the maintenance of ER homeostasis and execution of neural differentiation.

## Highlights

- FAF2 is required to regulate ER homeostasis in neural progenitor cells
- FAF2 knockout blocks differentiation of neural progenitor cells to neurons at the cell morphological level, but does not inhibit the mounting of transcriptional programs associated with neural differentiation.
- Excess ER stress due to FAF2 knockout contributes to blocked neural differentiation.

## Introduction

Neural progenitor cells are an essential component of the developing embryonic central nervous system. These cells must divide to expand the progenitor pool while also gaining specificity along their developmental trajectory to ultimately form the great diversity of neuronal subtypes and differentiate into mature neurons. Cell fate decisions are driven by a myriad of cell intrinsic and extrinsic factors (Bonnefont and Vanderhaeghen, 2021; Wang et al., 2025). These factors can be instructive, directing cell fate down a specific trajectory, or permissive, allowing cells to respond to the instructive cues (Tatapudy et al., 2017). Imbalance of these factors and failure of neural progenitor cells to execute the correct cell fate decisions can be disruptive to the generation of the nervous system. Thus, it is critical to gain a wholistic model of the types of cues that govern neural progenitor cell fate decisions.

Morphogen signaling and downstream transcription factor networks are instructive cues that have been well studied in neural progenitor cell fate. Removing these factors inhibits the cell from mounting the necessary cascade of signals to commit the cell toward a specific fate. Permissive cues provide the correct cellular environment to respond to instructive cues and carry out the cell fate changes. Permissive cues include processes that support cell stability and survival such as cell homeostasis and metabolism. While there is some evidence that permissive cues are necessary to support cell fate decisions in neural progenitor cells (Tatapudy et al., 2017), the links between cell homeostasis and the ability to respond to and carry out instructive cues and the homeostatic proteins that are integral to promoting differentiation is still understudied.

Endoplasmic reticulum (ER) stress can be a source of cellular instability (Rutkowski and Kaufman, 2004; Spencer and Finnie, 2020). The ER is the primary site of protein folding and quality control. When there is a shift in the ER toward more misfolded proteins, the ER enters a stressed state and employs strategies to regain homeostasis such as initiating the unfolded protein response (UPR). UPR serves to reduce the burden of misfolded proteins by reducing translation and increasing machinery that can remove misfolded proteins and chaperone the folding of new proteins (Hetz et al., 2020; Rutkowski and Kaufman, 2004; Zhang and Kaufman, 2006). The UPR functions through the activation of three receptors (ATF6, PERK, IRE1) which undergo modifications when the UPR chaperone, BIP, no longer binds to these receptors (Bertolotti et al., 2000; Hetz et al., 2020). The activated receptors induce transcription factor localization to the nucleus to turn on transcriptional programs aimed at reducing the burden of misfolded proteins.

ER stress and increased unfolded protein response are hallmark phenotypes in neurodegenerative disease and some neurodevelopmental contexts (Godin et al., 2016; Kawada et al., 2014; Murao and Nishitoh, 2017; Saito and Imaizumi, 2018; Wu et al., 2024). An increase in protein aggregates is often present in neurodegenerative diseases and can overwhelm the ER, sending the UPR into full affect (Saito and Imaizumi, 2018). In neurodevelopment, several neural tube defect models show increased ER stress as part of the pathobiology (Li et al., 2013; Yan et al., 2021). Although it is known that ER stress can modulate nervous system development, there is no consensus model of how ER stress can influence neural stem and progenitor cell fate decisions. In some cases, inducing the UPR by increasing ER stress can increase cell differentiation, in others too much ER stress can inhibit differentiation, and in some cases differentiation can proceed but with defects in neuronal maturation (Godin et al., 2016; Kawada et al., 2014; Vásquez et al., 2022). Several ER-stress associated proteins such as Sel1L, Hrd1, and ATF4 have been studied in the context of neural stem cell fate decisions and shown to play critical roles (Frank et al., 2010; Kawada et al., 2014; Saito et al., 2023). Even so, there is still limited information of the role that homeostatic proteins play in cell fate decisions. The goal of our study is to test the context-specific effect of ER stress on embryonic neural progenitor cell fate through the investigation of a homeostasis protein.

**FIGURE 1:**
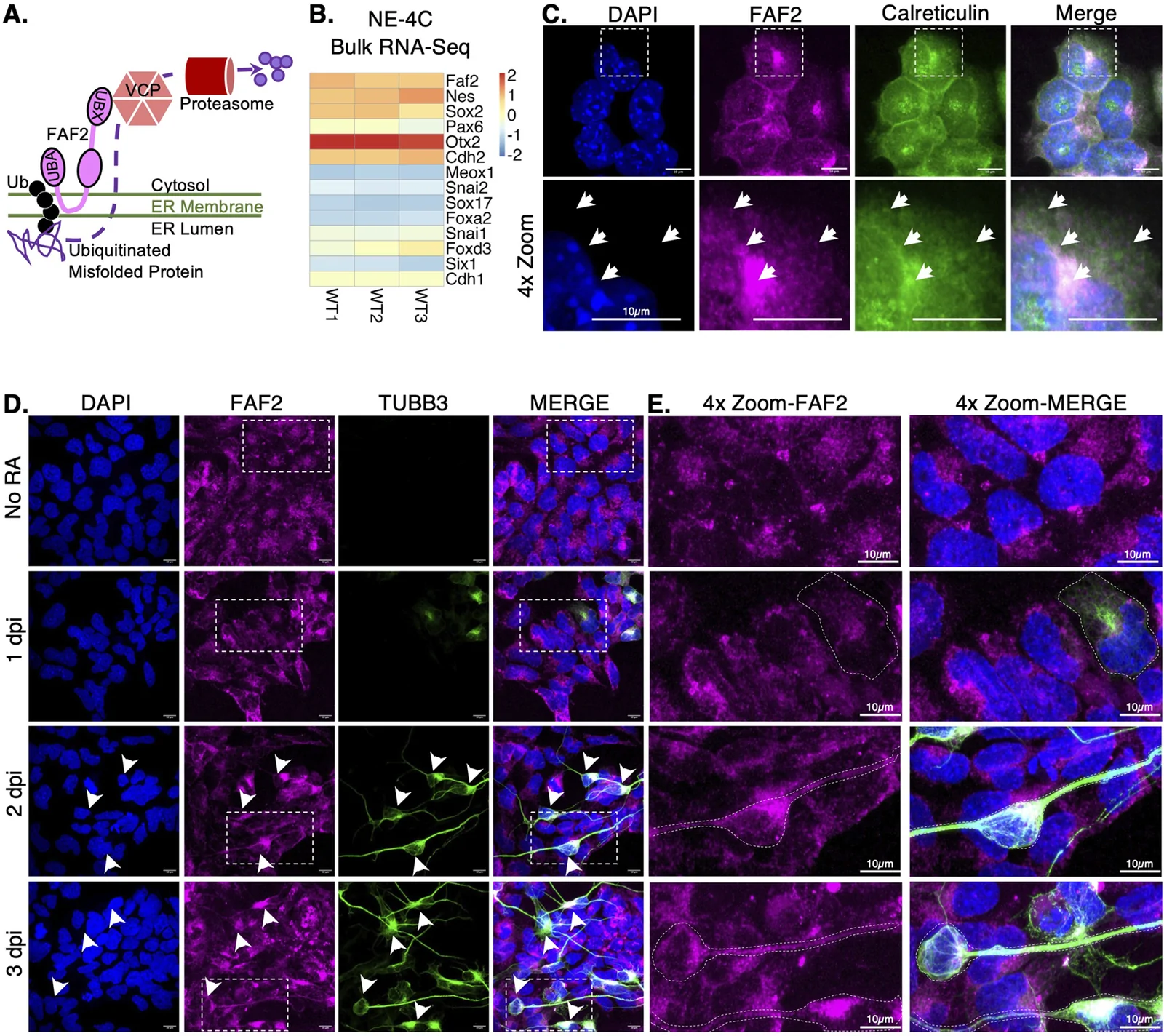
FAF2 is expressed in mouse embryonic neural progenitor cells. **(A)** Schematic of FAF2 domain structure and role in ER-regulated degradation of misfolded proteins. UBA: Ubiquitin-associated domain; UBX:ubiquitin regulatory x domain; Ub: ubiquitin. **(B)** Heatmap showing relative expression of genes that mark the three germ layers derived from bulk RNA-Seq of individual replicates of wildtype NE-4C cells. **(C)** Immunofluorescence of wildtype NE-4C cells against FAF2 (magenta) and Calreticulin (ER marker in green). Cells were counterstained with DAPI to visualize the nucleus. Top panel shows field of cells, bottom panels show 4x-digital zoom of dashed box. Arrows show examples of FAF2 and CALR overlap. **(D)** Immunofluorescence time-course of retinoic acid (RA) induced neural differentiation in wildtype NE-4C cells against FAF2 (magenta) and TUBB3 (neuronal marker in green). Arrows show differentiated neurons. Dpi = days post-induction. **(E)** 4x-digital zoomed images from dashed boxes in (D). Dotted lines show TUBB3 expression domains for individual cells.

Neural tube closure and neural tube defects are our primary area of interest and the Knockout Mouse Project identified that mouse embryos with a knockout of Fas-associated factor 2 (FAF2, UBXD8, ETEA) have neural tube defects. Neural tube closure requires tight regulation of neural progenitor cell fate decisions. FAF2 is a protein involved in homeostasis; thus we sought to investigate functions of FAF2 in neural progenitor cells that shed light on possible mechanisms underlying the neural tube defect phenotype. FAF2 is an ER membrane protein that functions as an adaptor to extract misfolded proteins from the ER to the cytoplasm for degradation (Mueller et al., 2008). FAF2 contains a ubiquitin associated (UBA) domain which identifies ubiquitinated proteins and a ubiquitin regulatory x (UBX) domain which recruits the ATPase, p97/VCP, to extract the ubiquitinated protein for proteasomal degradation (Christianson et al., 2011; Mueller et al., 2008; Zheng et al., 2022) (Figure 1A). FAF2 also modulates lipid biology by targeting proteins involved in lipid synthesis and lipid droplet regulation (Ganji et al., 2023, p. 97; Olzmann et al., 2013; Suzuki et al., 2012) and FAF2 is important for regulation of peroxisome homeostasis (Kim et al., 2025). Although these functions have been studied across several different cell types (HeLa, HEK293, U2OS, hepatocytes, adult neurons (Huda et al., 2025; Van Alstyne et al., 2025; Zheng et al., 2022), the function of FAF2 has not been studied in the context of stem cells, and specifically neural stem and progenitor cells. Here we establish FAF2 as a homeostasis protein in embryonic neural progenitor cells that is required for neuronal differentiation.

## Materials and methods

### NE-4C cell line

NE-4C cells were originally derived from E9 mouse embryonic brain (Schlett and Madarász, 1997). Our stocks were purchased from ATCC (CRL-2925). Cells were grown at 37 °C and 5% CO_2_ conditions in MEM (Gibco 41090101) supplemented with 10% FBS, 1xMEM nonessential amino acids (Gibco 11140050), 1xGlutaMax (Gibco 35050061), and 1% penicillin-streptomycin (Gibco 15070063). The NE-4C cell line was routinely tested for *mycoplasma* contamination.

### Genome editing using CRISPR/Cas9

The CRISPR-Cas9 DNA editing method to generate the *Faf2* knockout cell line was derived from (Zocher and Kempermann, 2021). Two sgRNAs were used to create a 700bp deletion to remove exon 4 of *Faf2* and create an early stop codon We used sgRNA sequences (CCC​ACT​GCG​ATG​ACC​ATG​CA; CGA​TCA​TAA​CAC​CTT​CCA​CT) as was done to generate the same deletion in a *Faf2* knockout mouse model by the Knockout Mouse Project (Faf2em1(IMPC)Mbp) (Wilson et al., 2026) (https://www.mousephenotype.org/). To create sgRNA/Cas9 plasmids we used the pSpCas9(BB)-2A-Puro (PX459) V2.0 plasmid from Addgene (62988). This plasmid contains the Cas9 coding sequence, a puromycin resistance cassette and cloning region for an sgRNA. Individual plasmids were cloned for each sgRNA sequence by inserting cloning-adapted oligos purchased from IDT into the *BbsI* cloning site as detailed in (Zocher and Kempermann, 2021) (sgRNA1: F-caccCCCACTGCGATGACCATGCA/R-aaacTGCATGGTCATCGCAGTGGG; sgRNA2:F- caccCGATCATAACACCTTCCACT/R-aaacAGTGGAAGGTGTTATGATCG). Insertion of the sgRNA was verified by whole-plasmid sequencing.

To edit cells, NE-4C cells were transfected with both sgRNA plasmids using Lipofectamine 3,000 (ThermoFisher Scientific L3000015) 1 day after plating 200,000 cells in a 12-well plate. On day 2-post transfection, the media was replaced with selection media containing 3 μg/mL puromycin for 2–4 days. After puromycin selection, remaining cells were re-plated to a 10 cm plate and individual colonies were picked to a 24 well plate when colonies reached ∼100 cells. Colonies were expanded to maintain the stock and genomic DNA collected for PCR. PCR was performed using the following primers: Common forward TAC​AGT​GAG​TTC​TGG​AAC​AGC​CAG​G; WT-reverse CAA​GTA​ATA​ACC​CCA​TCC​AAG​CAG​G; Mutant-reverse TAA​CCT​AAG​CAT​CGC​CAG​GG. Colonies showing only one band at 525bp representing the deletion product (Common forward/Mutant-reverse primer) were then validated by Sanger sequencing.

### Knockdown cell line generation by shRNA

Two shRNA plasmids targeting *Faf2* were purchased from the University of Colorado Anschutz Functional Genomics Shared Resource (RRID: SCR_021987). Plasmids included the *Faf2* shRNA sequence and puromycin resistance gene for selection. NE-4C cells were transfected with two shRNA plasmids (TRCN0000191263: CGG​TTT​ACC​TAT​TAC​ACG​ATA; TRCN0000191032: CCA​TGA​CTT​CTT​ATT​CTC​CTT) by Lipofectamine 3,000. On day 2-post transfection, the media was replaced with selection media containing 3 μg/mL puromycin for 1 week. Remaining cells were kept as a poly-clonal line. To confirm random integration of the plasmids into the cells for stable expression of the shRNA, genomic DNA was collected and used for PCR. PCR products from a forward primer common to the U6 promoter of the shRNA plasmid (CAA​GGC​TGT​TAG​AGA​GAT​AAT​TGG​A) and reverse primer specific to the target sequence (sh023 GAG​AAG​GAG​AAT​AAG​AAG​TCA​TGG; sh263 TAT​CGT​GTA​ATA​GGT​AAA​CCG​CCG) at 250bp represent DNA specific to the introduced plasmids that was integrated into the cellular genome.

### Neuronal differentiation and small molecule treatments

NE-4C neuronal differentiation was induced by addition of 1 µM all-trans retinoic acid (RA) (Sigma-Aldrich # 302-79-4) for 24 h followed by incubation in growth media (Varga et al., 2008). To induce ER stress, NE-4C cells were treated with 0.01 µM Thapsigargin (Tocris 1138) for 24 h. Stressed cells were then either collected for immunofluorescence or thapsigargin media was replaced with differentiation media. To reduce ER stress, NE-4C cells were treated with 1 mM 4-phenylbutyric acid (4-PBA, Cayman Chemical 11323) for 24 h. For differentiation experiments, 4-PBA treated cells were then cultured in media containing 4-PBA and RA for 24 h.

### Immunofluorescence, imaging, and analysis

For immunofluorescence experiments, cells were plated directly on glass coverslips in cell culture plates. Following experimentation, cells were fixed on coverslips in 4%PFA for 15 min at room temperature, washed in 1xPBS and then permeabilized in 0.1% Triton in 1xPBS (PBST) for 15 min at room temperature. Cells were blocked in PBST containing 1% normal goat serum from 30 min at room temperature before addition of primary antibodies diluted in blocking solution. Primary antibodies were incubated overnight at 4 °C. Following primary antibody incubation, cells were washed with PBST, then secondary antibody (1:500) and DAPI diluted in PBST were added for 2 h at room temperature. Cells were then washed in 1xPBS before being mounted on glass slides with Prolong Gold Antifade (Invitrogen P10144). Cells were imaged with a Yokogawa CellVoyager™ CV1000 spinning disk scanner housed by the University of Colorado Light Microscopy Core Facility (RRID:SCR_018993). Primary antibodies used: TUBB3 (BioLegend PRB-435P rabbit, 1:1000); FAF2 (ThermoFisher 66629-1-IG mouse, 1:300); pH3 (Cell Signaling Technology 9714 rabbit, 1:100); BIP (CST 3177S rabbit, 1:300); CALR (Abcam ab2907 1:100 rabbit).

### Thioflavin T staining

Cells were plated on glass coverslips and allowed to grow to ∼70–80% confluence. Media was then replaced with cell culture media containing 10 μg/mL Hoechst to stain nuclei and 5 µM Thioflavin T (Cayman Chemical 32553) to stain protein aggregates for 30 min in the 37 °C, 5% CO_2_ incubator. Staining media was replaced with 1xPBS and coverslips were mounted on glass slides and immediately imaged on the Yokogawa CellVoyager™ CV1000 spinning disk scanner.

### Quantitative image analysis

For each imaging experiment, 5–10 fields of view were imaged per coverslip and experiments were repeated a minimum of three times using different cell passages. For FAF2 expression*,* thioflavin T and differentiation experiments, ImageJ was used to measure pixel intensity of the DAPI channel and experimental channel (FAF2, BIP, TUBB3, ThT) from thresholded images. Experimental channel intensities were normalized to DAPI area for a normalized intensity value. The normalized value was log2 transformed and means were calculated from fields of view per experiment. For proliferation experiments, the number of pH3 positive cells were counted and normalized to the number of nuclei identified by DAPI for five fields of view per biological replicate. The mean log2 values or pH3/cell for the fields of view per each biological replicate were used to statistically test for differences between conditions. For experiments with two conditions, a Student’s t-test was used to test for significance; for experiments with more than two conditions, a two-way Anova was run with a *post-hoc* Tukey test to derive pairwise p-values.

### Western blots

Cells were collected by trypsinization, pelleted at 4000 RPM for 5 min, and washed in PBS before pelleting again. Cell pellets were resuspended in RIPA buffer and lysed on ice for 30 min. Lysate was then spun down at max speed for 20 min at 4 °C and supernatant was taken. Cell lysates were quantified by Bradford assay. Lamelli buffer was added to protein lysates at a 1:1 ratio and boiled for 15 min.

15 µg total protein were loaded onto 7.5% polyacrylamide gels for anti-FAF2 blots and 4%–20% gradient polyacrylamide gels for all other blots and run at 200V for 30 min. Protein was then transferred to PVDF membrane using the BioRad Turbotransfer system. Membranes were blocked in 5% milk in TBST for 30 min, then primary antibody was added to the blocking solution and incubated overnight at 4 °C. Following washing, secondary HRP antibodies were added to blots in blocking solution at 1:2000 dilution and incubated at room temperature for 1 h. Blots were washed and visualized on a BioRad Chemidoc with ECL substrate. Primary antibodies used: TUBB3 (BioLegend PRB-435P rabbit, 1:10000); FAF2 (ThermoFisher 16251-1-AP rabbit, 1:1000); pH3 (Cell Signaling Technology 9714 rabbit, 1:500); BIP (CST 3177S rabbit, 1:1000); p-eIF2a (CST 3398S rabbit, 1:1000); p-IRE1 (Fisher PA1-16927 rabbit, 1:000); ATF6 (Novus NBP175478SS rabbit, 1:1000); GAPDH (rabbit, 1:10000).

Western blots were quantified in FIJI using the gel analysis tool. Values for each protein per sample were normalized to the corresponding GAPDH band intensity. Log transformed normalized values were statistically compared and averages of minimum three biological replicates shown on plots. Experiments with two conditions were statistically tested by Student’s t-test; experiments with more than two conditions were statistically tested by an Anova with a *post hoc* Tukey test for pairwise p-values.

### Bulk RNA-Seq preparation and processing

WT and knockout cells were collected from three independent passages and frozen as cell pellets. The “NO” condition (not induced) were collected without perturbations. The “RA” condition (retinoic acid induced) were treated for 24 h with RA followed by basal media until collection at 3 days post induction. Total RNA was then extracted from cell pellets and sent for mRNA library preparation by The University of Colorado, Anschutz Shared Genomics Core (RRID: SCR_021984) using the Universal Plus™ mRNA-Seq library preparation kit with NuQuant. Libraries were sequenced at a depth of 40 million paired end reads on an Illumina NovaSeq.

Fastq files were processed and returned as a counts table using the established NF-Core RNA-Seq pipeline (https://nf-co.re/rnaseq/3.22.2/) in bash (Ewels et al., 2020). We used the default pipeline which uses STAR for alignment and Salmon for quantification. Reads were aligned to Mus_musculus.GRCm39.109. The output counts were normalized using DESeq2 in R using the vst command. PCA plot was generated with DESeq2 command “PlotPCA”. We then used DESeq2 to perform differential expression analysis on desired comparisons (Love et al., 2014) (Full differential expression gene list in Supplementary Table S1). Volcano plots were generated using log10pvalue and log fold change results from differential expression analysis. Raw Fastq files and metadata are available on GEO (GSE338508) and SRA (PRJNA1494588).

### GSEA and gene ontology analysis

Gene set enrichment analysis was performed using the R package fgsea (Korotkevich et al., 2021). Genes were ranked and ordered by the Wald statistic which is an output of differential expression analysis in DESeq2. The ranked list was inputted into the function *fgsea* and the GO:BP database for *Mus musculus* was queried. Only significantly enriched pathways are reported. Full gene set enrichment analysis results in Supplementary Table S2.

Gene ontology (GO) analysis for transcription factor enrichment was performed utilizing the R package gProfileR (Raudvere et al., 2019). Genes that were significantly increased or decreased in expression between WT and KO cells were treated separately as inputs for analysis. The GO terms from source “TF” were investigated for transcription factor binding motifs. The genes that called each transcription factor were then used for GO analysis; the output of this analysis was investigated for GO:Biological Processes. Full gene ontology analysis results in Supplementary Tables S3,4.

## Results

### FAF2 is present in embryonic mouse neural progenitor cells

While it is known that FAF2 is present in the ER of other cell types including hepatocytes, HeLa cells, HEK cells, and adult neurons, the role of FAF2 during embryonic neural development is unknown. To study FAF2 function in the context of embryonic neural stem cells, NE-4C cells were used as a model system. NE-4C cells are a commercially available cell line derived from embryonic day 9 (E9) mouse brain and immortalized by deletion of p53 (Schlett and Madarász, 1997). To provide a baseline for our studies and as a general resource, we performed RNA-Seq of WT NE-4C cells. Gene expression from WT NE-4C cells show that the neural progenitor cell molecular identity is preserved as revealed by higher expression of neural progenitor marker genes (*Nes, Sox2, Pax6, Otx2, Cdh2)* relative to genes that represent other cell types (*Meox1, Snai2, Sox17, Foxa2, Snai1, Foxd3, Six1, Cdh1)* (Figure 1B). *Faf2* is also expressed in neural progenitor cells and immunofluorescence confirmed FAF2 protein expression in NE-4C cells. As in other cell types, FAF2 has perinuclear localization and colocalizes with Calreticulin (CALR), which indicates its localization to the endoplasmic reticulum (Figure 1C).

### FAF2 protein is present during neural differentiation

Neural progenitor cells are specialized cells that must balance proliferation and differentiation to expand the progenitor pool while also producing new neurons. NE-4C cells are capable of differentiation by induction with All-Trans Retinoic Acid (RA) (Schlett and Madarász, 1997; Varga et al., 2008), thus we tested if FAF2 protein expression or localization differs between neural progenitor cells and neurons. For differentiation experiments, cells were treated with retinoic acid (RA) for 24 h and then RA was washed out and chased with growth media. Cells were collected to visualize expression of FAF2 and TUBB3, an early neuronal marker, over the 3 days post induction (dpi). FAF2 expression was similar in cycling neural progenitor cells and differentiating neurons (Figure 1D) with FAF2 polarized to one side of the nucleus and found in the cell bodies of neural progenitors and neurons. FAF2 protein was also found in the neurites of differentiating neurons (Figures 1D,E). The observed stable expression of FAF2 during differentiation was also supported by RNA-Seq expression data between cells that were not treated with RA and cells that were induced with RA and collected on day 3 post induction—*Faf2* mRNA expression was not significantly different between conditions (Supplementary Table S1).

### Loss of FAF2 results in ER stress signature

**FIGURE 2:**
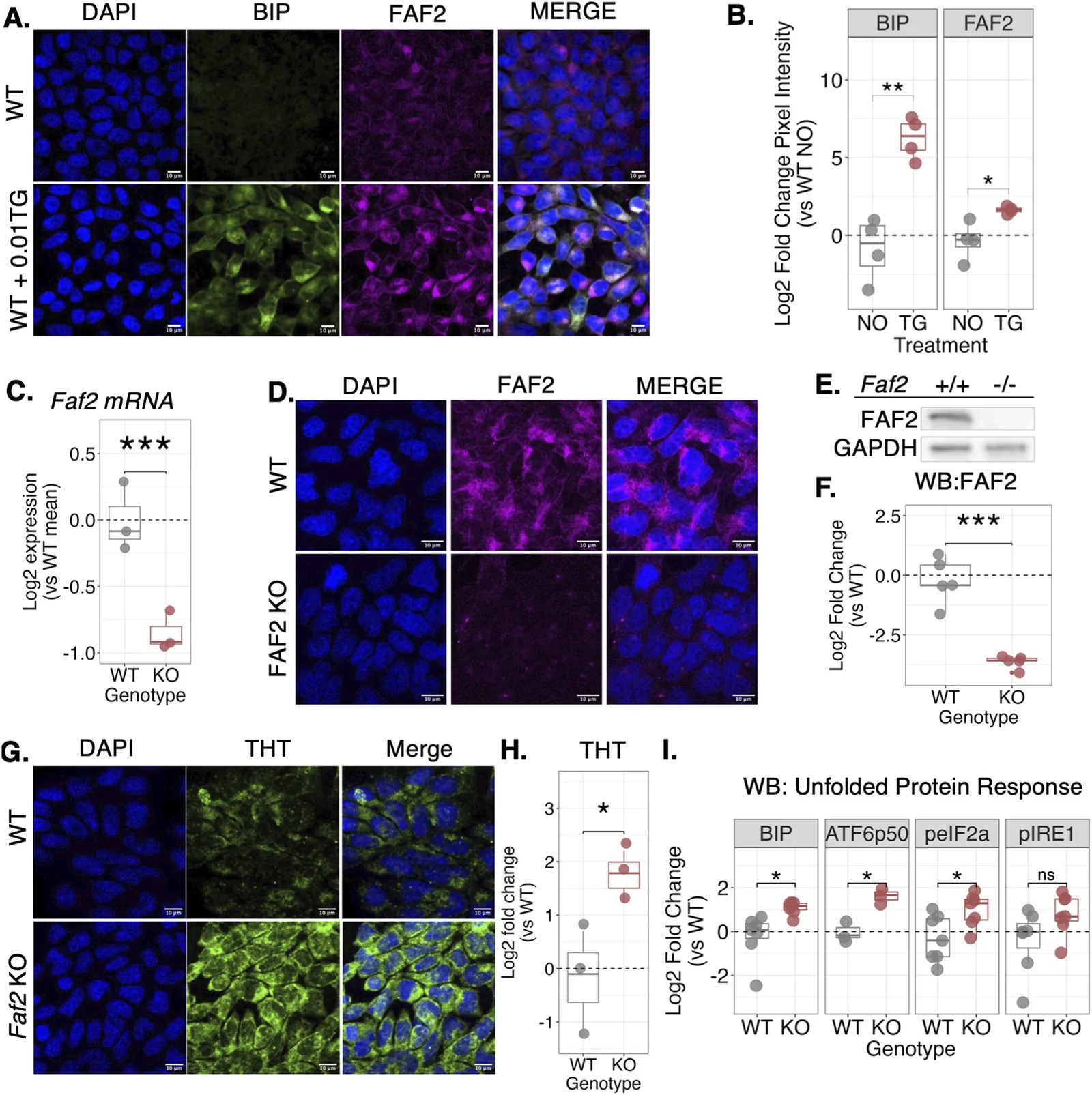
Faf2 knockout results in increased ER stress signature in neural progenitor cells. **(A)** Immunofluorescence against BIP (ER stress marker in green) and FAF2 (magenta) in untreated and Thapsigargin (TG) treated wildtype cells. **(B)** Quantification of pixel intensity of BIP and FAF2 as in (A). Each condition shows n = 4 biological replicates. **(C)** mRNA expression of *Faf2* in WT and *Faf2 KO* cells from bulk RNA-Seq data. **(D)** Immunofluorescence images of FAF2 (magenta) in WT and *Faf2 KO* cells. **(E)** Western blot of WT and *Faf2 KO* cells to assess FAF2 levels compared to GAPDH. **(F)** Quantification of western blots as in (E). n = 5 biological replicates. **(G)** Immunofluorescence images of thioflavin T (THT) staining (marker of misfolded proteins) of WT and *Faf2 KO* cells. Cells were counterstained with DAPI. **(H)** Quantification of THT pixel intensity from (G). **(I)** Quantification of western blots for unfolded protein response proteins. Representative western blot shown in Supplementary Figure S1D. *p < 0.05, **p < 0.01, ***p < 0.001.

FAF2 is critical for modulating ER stress and promoting cell homeostasis in other cell types, however this role has not been investigated in neural stem cells. To test if FAF2 protein expression is responsive to ER stress, NE-4C cells were treated with Thapsigargin (TG) to induce ER stress. Thapsigargin is a small molecule that depletes the ER of calcium, leading to disrupted protein folding (Li et al., 1993). Cells were treated with thapsigargin for 18 h and fixed for immunofluorescence with BIP, a chaperone of the unfolded protein response and used as an indicator of ER stress (Sicari et al., 2020). Thapsigargin treated cells had significantly more BIP pixel intensity than untreated cells (p = 0.002) (Figures 2A,B). FAF2 expression was also significantly increased in Thapsigargin treated cells (p = 0.045) indicating FAF2 protein expression in neural progenitor cells is responsive to acute ER stress (Figures 2A,B).

To test the hypothesis that FAF2 is required to reduce ER stress in neural progenitor cells, we generated a *Faf2* knockout line using CRISPR/Cas9 mediated exon 4 deletion. We identified successful deletion of exon 4 followed closely by a premature stop codon by PCR and Sanger sequencing (Supplementary Material 1A,B). *Faf2* mRNA expression was significantly reduced in KO cells (p = 0.00021) (Figure 2C). The FAF2 protein has several functional domains necessary to perform its role as an adaptor to extract misfolded proteins from the ER for proteasomal degradation. Exon 4 overlaps with the hydrophobic domain necessary to anchor FAF2 to the ER membrane and the early stop codon is predicted to terminate translation in exon 5 which would exclude the downstream UAS and UBX domains (Supplementary Material 1C). Without the hydrophobic domain, FAF2 will not anchor to the ER and without the UBX domain, FAF2 will not be able to recruit p97/VCP which is required for protein dislocation (Olzmann et al., 2013; Suzuki et al., 2012). FAF2 protein expression was successfully knocked out from NE-4C deletion cells relative to WT cells as shown by immunofluorescence and western blot (p = 0.00089) (Figures 2D–F).

The *Faf2 KO* cell line was used to investigate whether the levels of various ER homeostasis markers changed in response to a loss of FAF2. FAF2 is important for the clearance of misfolded proteins and therefore it reduces the buildup of protein aggregates. To test the hypothesis the *Faf2* KO would lead to a buildup of protein aggregates, protein aggregates were visualized by Thioflavin T (ThT) staining in WT and KO cells. Thioflavin T is a small molecule dye that has fluorescent properties when bound to beta-sheets present in protein aggregates; thus its incorporation and imaging has been used as a way to monitor the buildup of misfolded proteins and ER stress (Beriault and Werstuck, 2013). Indeed, KO cells had significantly more ThT pixel intensity than untreated WT cells (p = 0.049), suggesting that *Faf2* KO results in an increase in protein aggregation (Figures 2G,H).

Protein aggregation in the ER can trigger the unfolded protein response (UPR). The UPR is made up of three signaling branches that can be detected at the protein and transcription level (Sicari et al., 2020). Each branch (ATF6, PERK, IRE1) signals through receptors and corresponding activated transcription factors to drive expression of genes responsible to resolve the conflict. To test if the KO cells showed a signature of the UPR, western blot was used to detect protein expression of markers of the UPR. BIP was significantly expressed in KO cells (p = 0.0163) suggesting an overall engagement of the UPR (Figure 2I, Supplementary Material 1D). To evaluate the three branches, antibodies against ATF6, p-eIF2a/PERK, and p-IRE were used. ATF6p50 and p-eIF2a were significantly increased in KO cells (p = 0.011, p = 0.023) suggesting engagement of the ATF6 and PERK branches of the UPR (Figure 2I, Supplementary Material 1D). p-IRE1 also showed increased expression, although this was not statistically significant (Figure 2I, Supplementary Material 1D).

### Faf2 knockout cells show transcriptional signature of ER stress

**FIGURE 3 F3:**
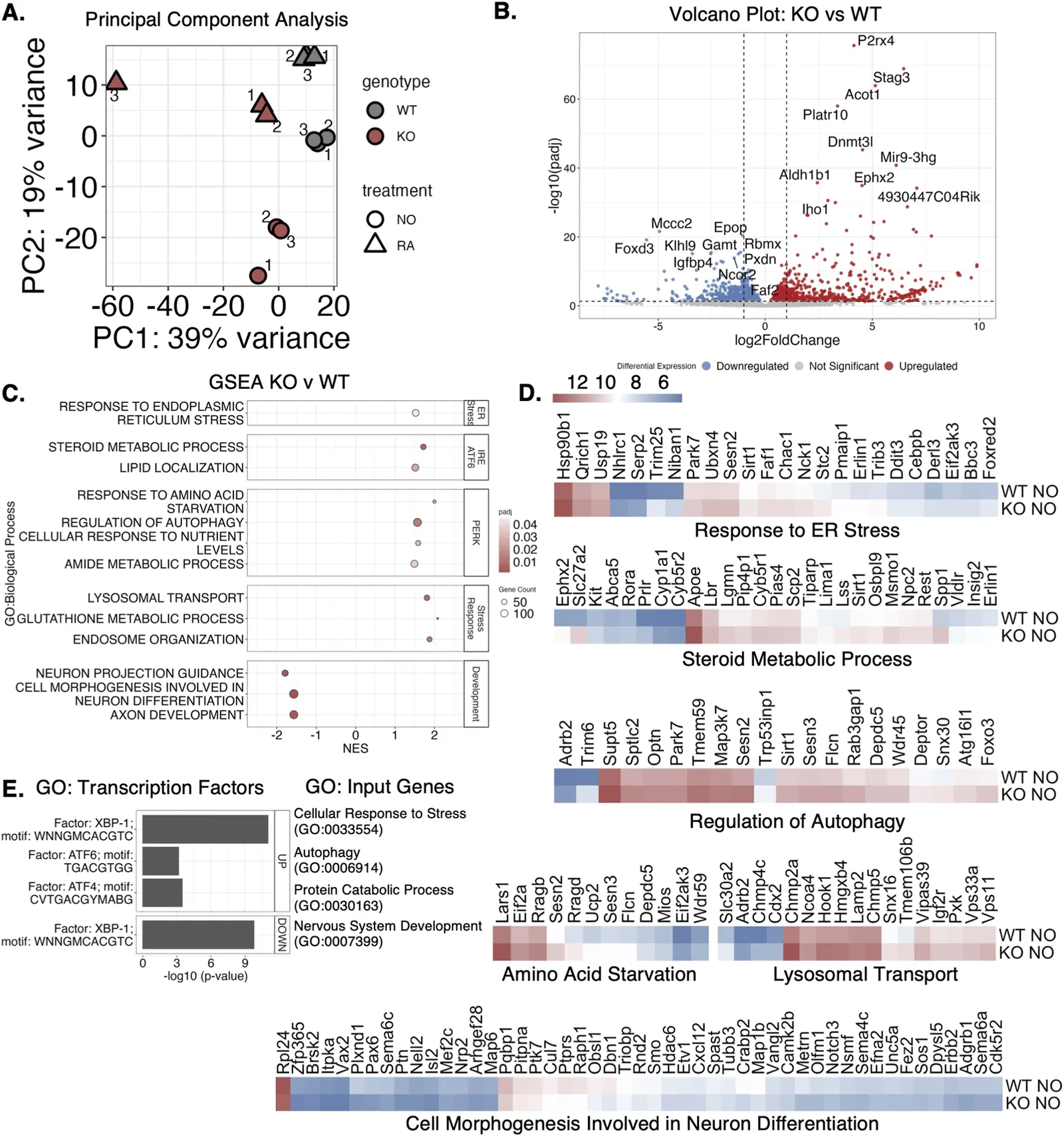
RNA-Seq reveals transcriptional signature of ER stress in FAF2 KO cells. **(A)** PCA plot of WT and KO samples that were untreated or 3 days post RA-treated. **(B)** Volcano plot of differentially expressed genes between untreated WT and KO cells. Top 10 differentially expressed genes are labeled. **(C)** Gene set enrichment analysis from bulk RNA-Seq of WT and *Faf2* KO cells. Graph shows representative terms related to cellular stress. Full list of terms can be found in the supplement. **(D)** Heatmaps of genes that are significantly differentially expressed between KO and WT cells which are part of the gene set that identifies enriched pathways in (C). **(E)** Gene-ontology analysis results for transcription factor calls between WT and KO cells. “UP” represents transcription factors called from upregulated genes and “Down” represents transcription factors called from downregulated genes.

To test if the KO cells showed a transcriptional signature of the UPR, RNA-Seq was used as an unbiased means to profile the transcriptome. WT and KO cells that were untreated (NO) or RA induced and collected at 3 days post induction (RA) were transcriptionally profiled by RNA-Seq. Principal component analysis (PCA) of the samples demonstrated a separation of samples by genotype, independent of treatment; within genotype, samples showed cleared grouping by treatment (Figure 3A). In PCA space, all samples within genotype and treatment groups had little variance except for KO + RA, biological replicate 3 cells (Figure 3A). Differential expression analysis between KO and WT cells revealed 1048 significantly upregulated genes and 1003 significantly downregulated genes (Supplementary Table S1). The volcano plot in Figure 3B summarizes the magnitude and significance of differentially expressed genes and highlights the top 10 differentially expressed genes ranked by adjusted p-value.

Differentially expressed genes between WT and KO cells were scored using the Wald statistic in DESeq2. Using all scored genes regardless of significance, we ran a gene set enrichment analysis (GSEA) to identify overrepresented pathways. Pathways that were positively enriched in KO cells included terms representative of all three UPR branches (IRE1/ATF6: “Steroid Metabolic Process”, “Lipid Localization; PERK “Response to Amino Acid Starvation”, “Regulation of Autophagy”, “Cellular Response to Nutrient Levels”, “Amide Metabolic Process”) (Figure 3C, full GSEA results in Supplementary Table S2). Significant differentially expressed genes between KO and WT samples were identified from the collection of genes driving representative enriched pathways (Figure 3D). Genes such as Ubxn4, Erlin1, Derl3, that drive the term “Response to Endoplasmic Reticulum Stress” are involved in ER-associated degradation and interact with FAF2 (Suzuki et al., 2012; Wolf et al., 2021). *Ephx2,* which is a driver of “Steroid Metabolic Process” (Figure 3D) is critical for lipid metabolism and is one of the top 10 upregulated genes in KO cells relative to WT cells (Figure 3B). GSEA identified the term (“Response to Amino Acid Starvation”) with a key upregulated gene*, eIF2a*, which complements our western blot analysis demonstrating upregulated PERK signaling by increased p-eIF2a (Figures 2I,3D, [Fig F3]). Pathways that were negatively correlated with the KO cells represented processes having to do with neural development and differentiation (“Neuron Projection Guidance”, “Cell Morphogenesis Involved in Neuron Differentiation”, “Axon Development”) (Figure 3C). The term “Cell morphogenesis involved in neuron differentiation” included genes involved in Shh signaling (*Smo)*, Notch signaling (*Notch3)*, Wnt-PCP signaling (*Vangl2)* (Figure 3D).

Given the gene sets that were enriched in KO cells, we next asked if relevant UPR-associated transcription factor motifs were also enriched. Using gProfiler and the TRANSFAC database, we identified predicted transcription factor binding motifs enriched in KO cells. In this analysis, the input genes represent differentially expressed target genes of transcription factors. These genes have been annotated with the inclusion of transcription factor binding sites within 1 kb of the transcription start site (Raudvere et al., 2019). Genes that showed increased expression in the KO cells revealed ATF4 (p = 0.00028), ATF6 (p = 0.021, p = 0.027), and XBP1 (p = 3.14e-9, p = 0.0026) binding motifs (Figure 3E). ATF4 or ATF6 binding motifs were not represented in genes that were downregulated, however XBP1 (p = 1.98e-7, p = 0.0007) motifs were called out for these genes (Figure 3E). gProfiler was used to identify gene ontology terms from the discrete set of genes which identified each transcription factor. The target genes of ATF4, ATF6, and XBP1 that were significantly increased in KO cells represented stress response terms consistent with the UPR pathways represented by each transcription factor (Figure 3E; Supplementary Figure S1). In contrast, the target genes that called XBP1 and were significantly decreased did not represent stress response genes, but instead called terms involved with developmental processes including the term “nervous system development” (Figure 3E; Supplementary Figure S2, full Gene ontology results in Supplementary Table S2). Overall, our data provides evidence that knocking out FAF2 results in a signature of ER stress, thus demonstrating a requirement for FAF2 to maintain ER homeostasis in NE-4C neural progenitor context.

### FAF2 is required for neural differentiation

**FIGURE 4:**
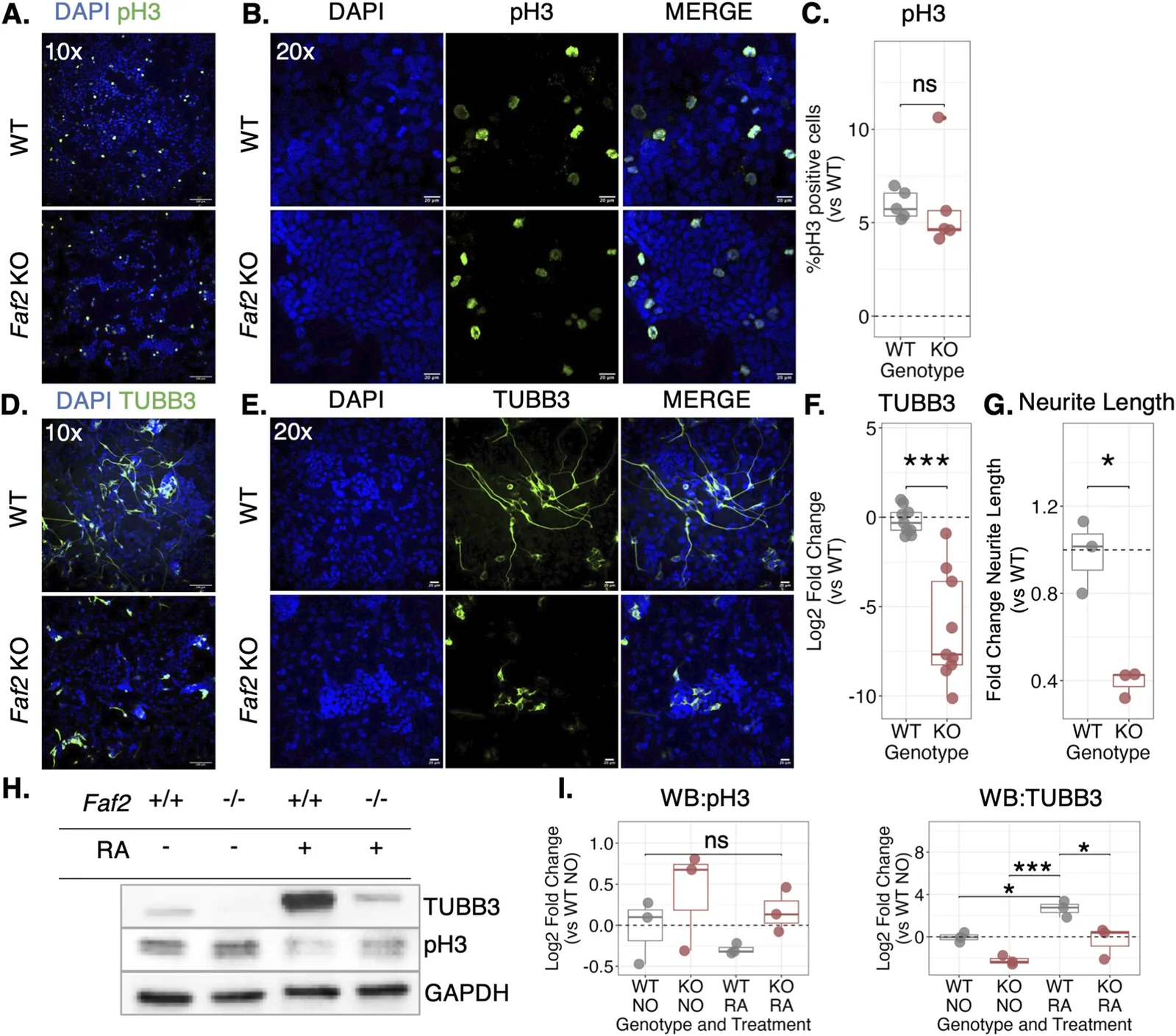
FAF2 is required for neural differentiation of NE-4C cells. **(A)** Immunofluorescence images against pH3 in WT and *Faf2 KO* cells at ×10 magnification, counterstained with DAPI. **(B)** Representative field of view at ×20 magnification of the same samples in (A). **(C)** Quantification of the percent pH3 positive cells per field of view. ×20 objective was used for collecting data for quantification. **(D)** Immunofluorescence images against TUBB3 in WT and *Faf2 KO* cells at ×10 magnification, counterstained with DAPI. **(E)** Representative field of view at ×20 magnification of same samples in **(D)**. **(F)** Quantification of pixel intensity of TUBB3 relative to DAPI channel in **(E)**. **(G)** Quantification of neurite length in images from (E). ×20 objective was used for collecting data for quantification in **(F,G)**. **(H)** Western blot against TUBB3, pH3, and GAPDH in WT and *Faf2 KO* cells that were untreated or treated with RA to induce differentiation. **(I)** Quantification of pH3 and TUBB3 bands from western blot as in (H). n = 3 biological replicates. Treatments: NO = untreated, RA = treated with all-trans retinoic acid. *p < 0.05, **p < 0.01, ***p < 0.001.

Since knocking out FAF2 showed a robust effect on ER stress modulation in neural progenitor cells, we next wanted to investigate if neural progenitor functions were sensitive to loss of FAF2. To test if cell proliferation rates were altered in KO cells, immunofluorescence and western blots against phospho-histone 3 (pH3) were used in WT and KO cells. Calculating the percent of pH3 positive cells per region of interest showed no significant difference between WT and KO cells, which was validated by western blot analysis of pH3 in whole protein lysates (Figures 4A–C,H,I).

We next tested the impact of the FAF2 knockout on neural differentiation. WT and KO cells were induced to differentiate with RA and fixed for immunofluorescence for Tubulinb-3 (TUBB3), a neuronal marker at 3 days post induction. KO cells had significantly reduced TUBB3 fluorescent intensity compared to WT cells (p = 0.00029) indicative of inhibited differentiation (Figures 4D–F). The blocked TUBB3 expression was also validated in whole protein lysates by western blot (WT + RA/KO + RA, p = 0.015)) (Figures 4H,I). Additionally, neurite lengths of the few cells that did express TUBB3 in KO cells were significantly shorter when compared to WT neurons (p = 0.016) (Figure 4G). These experiments were repeated using a NE-4C knockdown line generated by transfecting two shRNAs and selecting with puromycin for cells that integrated the shRNA for stable expression. The shFAF2 line was kept as a polyclonal line. When the shFAF2 line was treated with RA, we also found that differentiation was blocked (Supplementary Figure S3).

**FIGURE 5:**
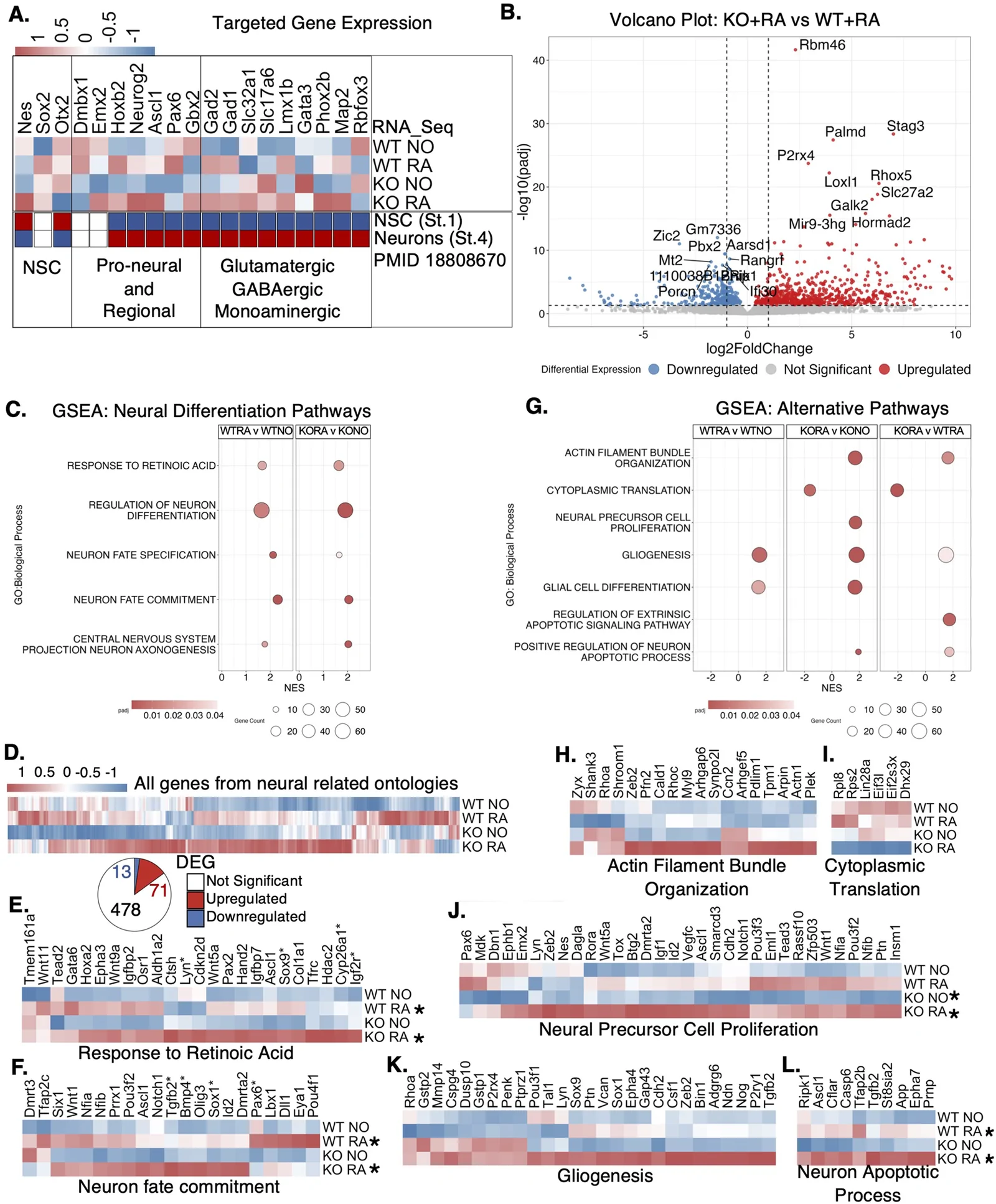
Faf2 KO cells can mount transcriptional response to differentiation induction by retinoic acid. **(A)** Marker genes to transcriptionally monitor differentiation of NE-4C cells. “NSC (ST.1)” and “Neurons (St. 4)” refer to stage of NE-4C differentiation and colors represent expected expression pattern as seen in (Varga et al., 2008). **(B)** Volcano plot of 3 days post induction retinoic acid treated WT and KO cells. Top 10 differentially expressed genes are labeled. **(C)** Select GSEA results representing neural differentiation in WT and KO comparisons between untreated and 3 days post RA-induced cells. **(D)** Gene expression from all genes that call enriched neural pathways from analysis in (C). Pie chart represents the break down of genes in the heatmap by differential expression status. **(E,F)** Gene expression of genes that call enriched pathways from (C) between untreated and RA-treated cells. * signifies significant differential expression between WT RA and KO RA conditions. **(G)** Select GSEA results of enriched paths demonstrating potential alternative cell fates for KO cells. **(H-L)** Representative gene expression from select pathways shown in (G). All genes in these heatmaps are significantly differentially expressed between the two *-denoted samples.

The data above of reduced TUBB3 expression and disrupted neurite elongation indicates that knocking out FAF2 results in reduced differentiation. However, it is unclear what mechanism underlies this effect. One hypothesis is that the KO cells cannot mount a transcriptional response to promote neural differentiation when treated with RA. Bulk RNA-Seq of RA-induced cells (“RA”) that were collected 3 days post induction and untreated cells (“NO”) from WT and KO backgrounds was used to test if genes related to neural differentiation were responsive to induction. We first used a targeted approach to assess the progress of differentiation in WT and KO cells using marker genes defined in (Schlett and Madarász, 1997; Varga et al., 2008). These markers include genes related to stemness (*Otx2*), pro-neural (*Ascl1, Neurog2),* regional-specificity (*Dmbx2*, *Emx2, Hoxb2, Pax6, Gbx2),* and mature neuronal subtypes (*Gad2, Gad1, Slc23a1, Slc17a6, Lmx1b, Gata3, Phox2b, Rbfox3, Map2)*. Many of the marker genes in the WT cells follow the trends established in (Varga et al., 2008), including decreased *Nes* and *Otx2* expression and increased pro-neural, regional, and mature neuronal gene expression (Figure 5A). The KO cells also followed trends of differentiation suggesting they can mount a transcriptional response to retinoic acid (Figure 5A).

The transcriptional response to RA induction was also assessed using differential expression analysis and pathway enrichment analysis. First, differential expression analysis between KO + RA cells and WT + RA cells revealed 833 significantly upregulated genes and 484 significantly downregulated genes (Supplementary Table S1). Several of the most significantly increased genes were the same as in the untreated KO and WT comparison (*Stag3*, *P2rx4*, *Mir9-3hg*) (Figures 3B, 5B). Several of the top 10 downregulated genes have known functions in neurodevelopment (*Zic2, Pbx2, Porcn)* (Figure 5B). Next, within each genotype, differential expression analysis was used to score genes between RA and untreated conditions; the ranked genes were then used for GSEA regardless of statistical significance (Full GSEA results in Supplementary Table S2). Resulting terms from the WT and KO analyses were filtered by three criteria: 1) terms which were enriched in neurons relative to NPCs (normalized enrichment score (NES) > 0), 2) terms related to neural differentiation, and 3) terms that were present in both WT and KO analyses and met conditions 1 and 2. These select terms are reported in Figure 5C. Both WT and KO, RA-induced cells showed transcriptional signatures of differentiation with clear shifts in relative gene expression from untreated condition to RA condition (Figures 5D–G). KO cells had enrichment of genes that called terms such as “Cellular response to retinoic acid”, “Neuron fate specification”, “Neuron fate commitment”, and “Central nervous system projection neuron axonogenesis”. Several of the genes that identified these terms were significantly differentially expressed between WT RA treated cells and KO RA treated cells, however many were not (Figures 5D–F). Of the 562 genes that were used to identify the neural gene set enrichment analysis terms in WT and KO cells, 84 were differentially expressed (71 upregulated and 13 downregulated in KO cells) (Figure 5D). Genes that comprise the upregulated gene set include transcription factors and signaling-related genes that promote differentiation (ex. *Jag1, Dlk1, Tgfb2,)* as well as genes that actively promote cellular morphology of neurons *(ex. Sema3, Nptx1, Ptn)*. The downregulated genes include negative regulators of stemness (ex. *Hes6, Disp3, Notum)*. Taken together, this data indicates that instructive cues were initiated in KO cells in response to RA leading to a transcriptional response reflective of a neuronal fate change (Figure 5), however differentiation at a morphological level was blocked (Figure 4).

Although KO cells can mount a transcriptional response to RA, they fail to progress to neuronal morphology GSEA analysis identified several pathways that highlight molecular signatures that may underlie this deficit in RA induced KO cells (Figure 5G; Supplementary Table S2). KO cells may not be able to create the cytoskeleton network necessary to complete cell morphogenesis into neurons. “Actin Filament Bundle Organization” is enriched in KO + RA cells relative to KO cells and WT + RA cells indicating a need for transcription of actin-related genes (Figures 5G,H) (Djalali et al., 2005; Lalisse et al., 2018). One function function of the UPR is to attenuate translation and prioritize removing misfolded proteins in contrast to the need for increased translational capacity for neural differentiation (Blair et al., 2017; Hoye and Silver, 2021). GSEA comparing KO + RA relative to KO and WT + RA reveals a downregulation of genes that identify “Cytoplasmic translation” which may be inhibitory to differentiation (Figures 5G,I). “Neural precursor cell proliferation” is also positively enriched in KO + RA cells relative to untreated KO cells (Figures 5G,J). *Notch1, Wnt1, Pou3f2, and Igf1* were of the most significantly changed genes in this pathway. Furthermore, NE-4C cells are capable of glial differentiation following RA induction, however this typically occurs 10 days post induction (Varga et al., 2008). “Gliogenesis” and “Glial cell differentiation” were positively enriched in both WT + RA vs. WT and KO + RA vs. KO comparisons suggesting RA induced a glial transcriptional program regardless of genotype (Figure 5G). “Gliogenesis” was also enriched when comparing KO + RA to WT + RA cells (Figure 5G,K) with *Cspg4, Penk, and Gstp1* among the most significantly upregulated genes. Additionally, *P2rx4* was included in the “Gliogenesis” term and is among the top 10 upregulated genes in both KO vs. WT and KO + RA vs. WT *+ RA* cells. *P2rx4* is expressed in the nervous system and known to promote BDNF expression (Djalali et al., 2005; Lalisse et al., 2018). GSEA also revealed that terms related to apoptosis (ex. “Regulation of extrinsic apoptotic signaling pathway”, “positive regulation of neuron apoptotic process”) are enriched when comparing KO + RA to KO cells and KO + RA to WT + RA cells (Figure 5G,L). *Casp6* is a key differentially expressed gene in this pathway. While the transcriptional profiling suggests possible alternative cell fates and mechanisms of failed neuronal morphology, validation requires future experiments.

### Reducing ER stress is sufficient to rescue neural differentiation in Faf2 KO cells

**FIGURE 6:**
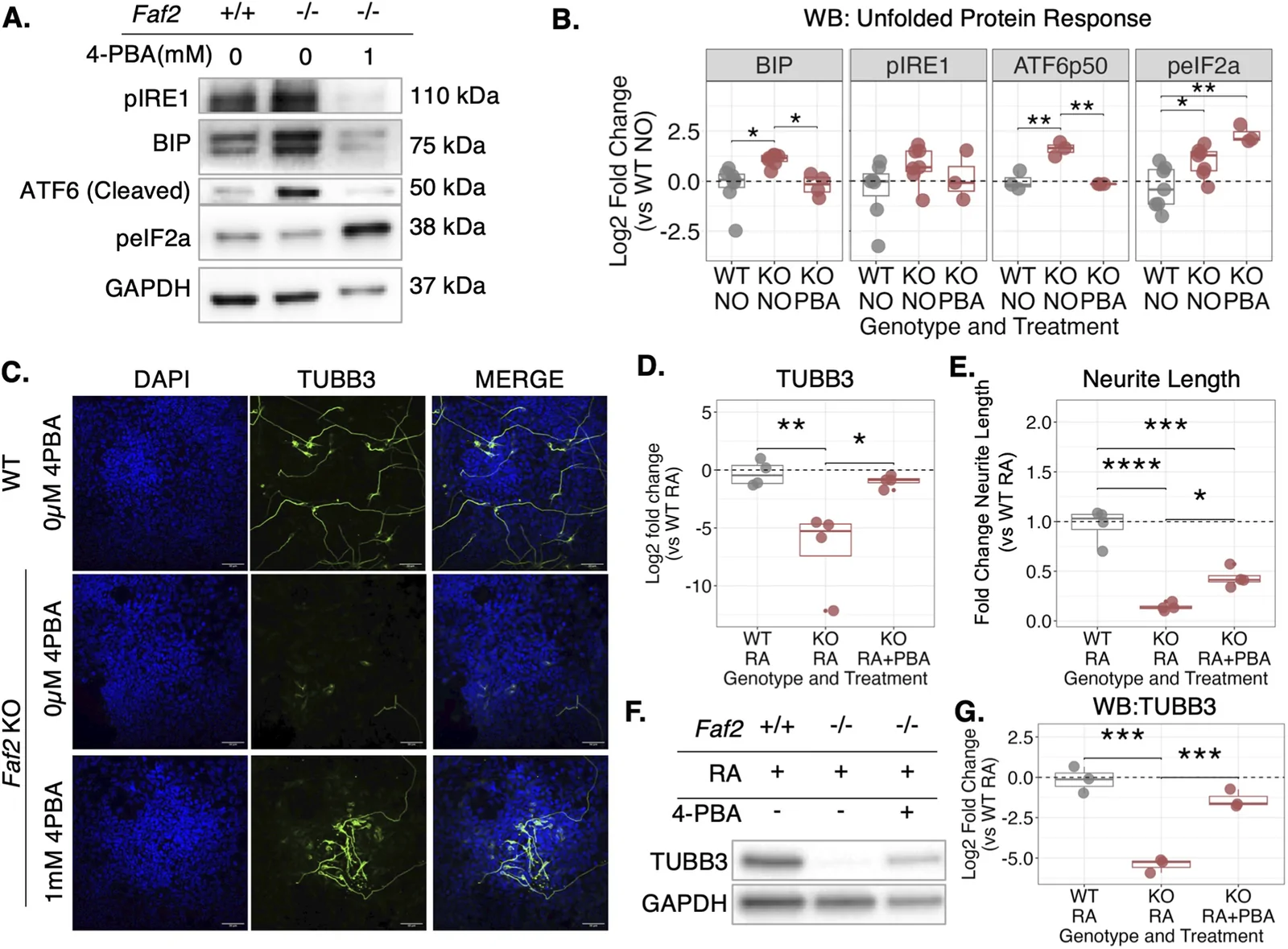
Reducing ER stress is sufficient to rescue differentiation in Faf2 KO neural progenitor cells. **(A)** Western blot of unfolded protein response proteins (p-IRE1 110kDa, BIP 75 kDa, ATF6 50kDa, p-eIF2a 38kDa, GAPDH 37kDa) in WT, untreated KO, and 4-PBA treated KO cells. **(B)** Quantification of western blots as in **(A)**. **(C)** Immunofluorescence against TUBB3 (green) in WT, untreated KO, and 4-PBA treated KO cells 3 days post-induction with RA. **(D)** Quantification of TUBB3 pixel intensity. **(E)** Quantification of neurite length. **(F)** Western blot against TUBB3 in WT, untreated KO, and 4-PBA treated KO cells that were induced to differentiate with RA. **(G)** Quantification of western blots as in (F). *p < 0.05, **p < 0.01, ***p < 0.001; n = 3 biological replicates.

Instructive transcriptional cues were initiated in KO cells in response to RA, however neuronal differentiation was not carried out at the cellular level. This data suggests that the KO of *Faf2* and resulting excess ER stress did not inhibit the ability of cells to respond to instructive cues. We thus hypothesized that ER homeostasis may be acting as a permissive cue and that increased ER stress restricted KO cells from carrying out the morphological aspects of neural differentiation. This hypothesis was tested by reducing ER stress in KO cells and assaying if this was sufficient to rescue differentiation. To reduce ER stress, KO cells were treated with the small molecule 4-Phenylbutyric Acid (4-PBA) for 24 h which acts as a chemical chaperone of protein folding (Kubota et al., 2006). In the 4-PBA treated KO cells relative to untreated KO cells, BIP and ATF6p50 protein expression were significantly reduced (p = 0.037, p = 0.0019), and p-IRE expression was reduced, although this latter result was not statistically significant (Figures 6A,B). In contrast, p-eIF2a expression was significantly increased in 4-PBA treated KO cells relative to untreated KO cells (p = 0.0017), the importance of which is addressed in the discussion (Figures 6A,B). These results indicate that ER stress can be reduced in KO cells with 4-PBA.

To test if reducing ER stress is sufficient to rescue differentiation, KO cells were treated with 4-PBA for 24 h prior to RA induction. KO cells treated with 4-PBA and collected 3 days post RA induction showed significantly increased TUBB3 intensity (p = 0.011) and longer neurites (p = 0.017) relative to untreated KO cells (Figures 6C–E). 4-PBA also rescued neural differentiation in the shFAF2 knockdown cells (Supplementary Material 3I,J). Western blot analysis of whole protein lysates also showed a statistically significant increase in TUBB3 expression in PBA treated KO cells (p = 0.00051) (Figures 6F,G). This evidence indicates that in the NE-4C cell context, excess ER stress resulting from knockout of FAF2 is inhibitory to neural differentiation. Together our data implicate FAF2 as a critical player in modulating ER stress to allow neural progenitor cells to retain their competence to undergo neuronal differentiation.

## Discussion

Our study establishes that FAF2 is expressed in embryonic neural progenitor cells and is required for ER homeostasis regulation and neural differentiation. *Faf2* is relatively highly expressed when compared to genes that specify neural progenitor identity. This is not surprising as it is a ubiquitously expressed homeostasis gene necessary for regulating cell stress. In NE-4C neural progenitor cells, FAF2 protein localized to the ER as in other cell types and did not change expression, localization or intensity from cycling to differentiated states.

We find that FAF2 is a critical regulator of neural progenitor ER homeostasis. ER stress induced by thapsigargin increases FAF2 protein expression in neural progenitor cells. This indicates that FAF2 expression is responsive to ER stress and is consistent with its role in the ER associated degradation response to remove misfolded proteins (Li et al., 1993; Rutkowski and Kaufman, 2004). Furthermore, FAF2 is critical for reducing the presence of misfolded proteins as knocking out *Faf2* led to an increase in thioflavin T positive protein aggregates. FAF2 knockout cells also showed an increased signature of the unfolded protein response as the PERK and ATF6 pathways were significantly enriched at mRNA and protein levels. The loss of FAF2 function disrupts the ER associated degradation response to remove misfolded proteins and stimulates the PERK pathway which inhibits new protein production and supports autophagic removal of misfolded proteins to rebalance cellular homeostasis. There is also a compensatory response to the disruption in ER associated degradation by transcription of genes that work in tandem with FAF2 to remove misfolded proteins.

Our *Faf2* loss of function experiments revealed that FAF2 is not required for neural progenitor cell proliferation but is required for neural differentiation. *Faf2 KO* cells had greatly reduced TUBB3 expression and fewer neurons, and those cells that did differentiate had much shorter neurites. Our original hypothesis was that *Faf2 KO* cells were unable to turn on or respond to instructive cues upon retinoic acid induced differentiation. However, RNA-Seq revealed that at a transcriptional level, genes involved in neural differentiation were expressed in a similar pattern compared to WT cells. The majority of genes that identified neurodevelopment related pathways were not significantly altered and the genes that were significantly up and downregulated in KO cells supported a program of neural differentiation. The relative changes in gene expression suggest KO cells are capable of responding to retinoic acid and ramping up transcription of genes related to differentiation and cellular morphogenesis of neurons, but morphological differentiation appears blocked. This data reveals that FAF2 is not required for interpretation of retinoic acid into downstream transcriptional programs but is needed to facilitate the conversion of those cues into the cellular behaviors reflective of neuronal differentiation such as neurite extension.

Transcriptomic analysis provided evidence that KO cells may retain progenitor character, die by apoptosis, or differentiate to glial cells. The analysis also suggests that KO cells cannot differentiate due to reduced protein translation and inability to modulate organization of actin filament network. During the differentiation process, progenitor cells that have not differentiated continue to divide and expand as a base layer underlying neurons. We continue to see this progenitor expansion as indicated by the field of DAPI-positive cells that are not TUBB3-positive (Figure 4A,B,D,E). We only see a measurable difference in TUBB3+ neurons between RA-induced WT and KO cells, thus it might be the case that cells die by apoptosis due to the sustained ER stress, cells take on glial fate, or the cells cannot complete cellular morphogenesis. While GSEA analysis identified glial differentiation terms, many of the genes that will identify glial terms also identify neuronal differentiation terms due to the sequential nature of differentiation in the NE-4C cells (Varga et al., 2008) and shared transcriptional programs (Sagner et al., 2021). Due to the stereotyped timeline of NE-4C differentiation into neurons and astroglia (the latter occurring days later), we do not suspect a profound fate switch in KO cells to gliogenesis. to be the case. Since we observe a transcriptional program in KO + RA cells that supports differentiation and mimics WT + RA cells, we favor the idea that blocking differentiation occurs at the cell morphogenesis level. Neuronal differentiation requires cytoskeleton rearrangement to form neurites (Luo, 2002). One hypothesis is that actin cannot be appropriately rearranged to complete morphological differentiation and feedback from this limitation can be observed in the upregulation genes that identify the term “Actin filament bundle organization”. Additionally, the PERK branch of the UPR can regulate Actin localization presenting a possible mechanistic connection between ER stress and actin rearrangement needed for differentiation (van Vliet et al., 2017). The RNA-Seq dataset presents several interesting directions to pursue and functionally validate.

A recent study showed that excess ER stress resulting from alcohol exposure has a similar phenotype in NE-4C cells: inhibition of differentiation but no effect on proliferation (Zhang et al., 2026). Moreover, in other neural stem and progenitor cell contexts, dysregulation of ER homeostasis can disrupt neural differentiation (Kawada et al., 2014; Murao and Nishitoh, 2017). Given that FAF2 is required to regulate ER homeostasis, we hypothesized that excess ER stress could underlie the inhibition of neural differentiation in the *Faf2 KO* cells. We tested this hypothesis by treating KO cells with an inhibitor of ER stress. While BIP, ATF6 and p-IRE1 responded in a way that suggests reduced stress in the *Faf2 KO* cells, p-eIF2a was increased. Usually, treatment of cells with 4-PBA will reduce the phosphorylation of eIF2a, however in our case it does not, instead it significantly increases. Increased p-eIF2a indicates that the PERK pathway is stimulated which inhibits new protein production and supports autophagic removal of misfolded proteins to rebalance cellular homeostasis. The phosphorylation of eIF2a specifically points to attenuated protein translation (Hetz et al., 2020). Global translation of proteins and the regulation of translation are critical for the cell fate change from neural progenitor cell to neurons (Blair et al., 2017; Hoye and Silver, 2021). We hypothesize that KO cells that are already experiencing disrupted homeostasis are sensitive to the global increase in translation that is needed to support neurogenesis. Our gene set enrichment analysis of KO-RA treated cells against WT-RA treated cells shows a reduction in expression of genes in KO cells related to translation processes (Figure 5G; Supplementary Table S2). Perhaps, p-eIF2a remains elevated to continue buffering translation and reducing misfolded protein rates, although future experiments are needed to test this idea. Regardless, 4-PBA treatment shows an overall indication of reduced ER stress and a partial rescue of neural differentiation as assessed by TUBB3 expression and neurite length. This demonstrates that the neural differentiation defect resulting from *Faf2 KO* is at least in part due to excess ER stress levels.

The partial rescue by the ER stress inhibitor suggests that FAF2 is critical for additional functions during neural differentiation. Lipid-related processes and oxidative stress-related processes were also enriched in *Faf2 KO* cells (Figure 3C,D). FAF2 adaptor function is critical for regulation of lipid synthesis (Ganji et al., 2023) and the maintenance of lipid droplets (Olzmann et al., 2013). While there are limited studies on the role of lipid droplet homeostasis in neural differentiation, it has been shown that reducing lipid droplet availability in adult neural stem cells can reduce neural differentiation (Ramosaj et al., 2021). Without FAF2, we hypothesize that lipid synthesis is reduced which could contribute to extra stress on neural progenitor cells and induction of transcriptional programs to increase lipid processes through the ATF6 branch of the unfolded protein response (Hetz et al., 2020). Furthermore, FAF2 can regulate lipid synthesis which is necessary for providing lipids to act as building blocks for cell membranes and to serve as signaling molecules. Studies show that treatment of neural progenitor cells with a variety of lipids can enhance neural differentiation and reducing lipid synthesis through knockout of genes such as *Fasn* can lead to a reduction in neural differentiation (Cabot et al., 2025; Gupta et al., 2025; Knobloch et al., 2013). Additionally, FAF2 is required to protect lipid droplets from being broken down by the lipase, ATGL (Olzmann et al., 2013); without FAF2, lipid droplets may be abnormally broken down, releasing triacylglycerols to the cytoplasm where they can be peroxidized and increase oxidative stress. Glutathione metabolism was an enriched term in KO cells and is a major pathway that the cell uses to combat oxidative stress (Wu et al., 2004). The case for oxidative stress in neural differentiation is complex and context specific. A neuroblastoma line (SHY-SHY cells) challenged with increased oxidative stress during neural induction showed no change in cell morphology and protein markers of differentiation but transcription was significantly altered (Khavari et al., 2020). In contrast, primary neural progenitor cells treated with hydrogen peroxide showed increased pro-neural gene expression and increased neural differentiation (Pérez Estrada et al., 2014). It is known that oxidative stress and ER stress can feedback on one another, thus reducing cellular homeostasis and stability (Cao and Kaufman, 2014). Future studies are needed to determine the relative contributions of these lipid and oxidative stress-related pathways in the cellular and molecular phenotypes of *Faf2 KO* cells.

Future experiments using different modalities to restore balance to the multiple homeostatic disruptions independently or at once will reveal if there are more critical homeostatic process for differentiation or if restoring the pathways can be synergistic. Additionally, re-introducing FAF2 and assaying the different pathways it regulates as well as differentiation will provide a more wholistic picture of FAF2 in differentiation. Nonetheless, our current evidence focused on the ER stress axis of FAF2 and supports the model that excess ER stress downstream of loss of FAF2 function is a key factor in the block of neural differentiation.

## Conclusion

Our data show that FAF2 is expressed in neural progenitor cells with a conserved function of reducing ER stress in the cell to help maintain homeostasis. *Faf2* loss of function neural progenitor cells show a signature of increased ER stress. *Faf2 KO* does not restrict the transcriptional response to retinoic acid induced differentiation, but neuronal morphology is blocked. Reducing ER stress in *Faf2 KO* cells is sufficient to rescue neural differentiation. Taken together, we have established FAF2 as a critical player in neural progenitor cells for regulating ER homeostasis to support cell competence to act on differentiation cues.

## Data Availability

Raw RNA-Seq files are publicly available on GEO (GSE338508) and SRA (PRJNA1494588).
