# Supplementary material for: Faf2 is required for neural differentiation in embryonic neural progenitor cells
Source: Front Cell Dev Biol. 2026 Aug 26;14:1935883. doi: 10.3389/fcell.2026.1935883 (PMC13553756; doi:10.3389/fcell.2026.1935883)
Supplement: Supplementary file 1 [file Supplementaryfile1.docx]

**Supplementary Appendix**

**Tables**

- Supplementary Table 1: Differential expression analysis results
- Supplementary Table 2: Gene set enrichment analysis full results
- Supplementary Table 3: Gene ontology Transcription Factor full results
- Supplementary Table 4: Gene ontology Transcription Factor input genes full results
